# Comparison of Strategies and Incidence Thresholds for Vi Conjugate Vaccines Against Typhoid Fever: A Cost-effectiveness Modeling Study

**DOI:** 10.1093/infdis/jix598

**Published:** 2018-02-12

**Authors:** Nathan C Lo, Ribhav Gupta, Jeffrey D Stanaway, Denise O Garrett, Isaac I Bogoch, Stephen P Luby, Jason R Andrews

**Affiliations:** 1 Division of Infectious Diseases and Geographic Medicine, California; 2 Division of Epidemiology, Stanford University School of Medicine, California; 3 Institute for Health Metrics and Evaluation, University of Washington, Seattle; 4 Typhoid Programs, Sabin Vaccine Institute, Washington, D. C; 5 Department of Medicine, University of Toronto, Washington, D. C; 6 Division of Internal Medicine, Toronto General Hospital, University Health Network, Toronto, Canada; 7 Division of Infectious Diseases, Toronto General Hospital, University Health Network, Toronto, Canada

**Keywords:** Typhoid fever, enteric fever, cost-effectiveness, mathematical modeling, vaccination, health policy

## Abstract

**Background:**

Typhoid fever remains a major public health problem globally. While new Vi conjugate vaccines hold promise for averting disease, the optimal programmatic delivery remains unclear. We aimed to identify the strategies and associated epidemiologic conditions under which Vi conjugate vaccines would be cost-effective.

**Methods:**

We developed a dynamic, age-structured transmission and cost-effectiveness model that simulated multiple vaccination strategies with a typhoid Vi conjugate vaccine from a societal perspective. We simulated 10-year vaccination programs with (1) routine immunization of infants (aged <1 year) through the Expanded Program on Immunization (EPI) and (2) routine immunization of infants through the EPI plus a 1-time catch-up campaign in school-aged children (aged 5–14 years). In the base case analysis, we assumed a 0.5% case-fatality rate for all cases of clinically symptomatic typhoid fever and defined strategies as highly cost-effective by using the definition of a low-income country (defined as a country with a gross domestic product of $1045 per capita). We defined incidence as the true number of clinically symptomatic people in the population per year.

**Results:**

Vi conjugate typhoid vaccines were highly cost-effective when administered by routine immunization activities through the EPI in settings with an annual incidence of >50 cases/100000 (95% uncertainty interval, 40–75 cases) and when administered through the EPI plus a catch-up campaign in settings with an annual incidence of >130 cases/100000 (95% uncertainty interval, 50–395 cases). The incidence threshold was sensitive to the typhoid-related case-fatality rate, carrier contribution to transmission, vaccine characteristics, and country-specific economic threshold for cost-effectiveness.

**Conclusions:**

Typhoid Vi conjugate vaccines would be highly cost-effective in low-income countries in settings of moderate typhoid incidence (50 cases/100000 annually). These results were sensitive to case-fatality rates, underscoring the need to consider factors contributing to typhoid mortality (eg, healthcare access and antimicrobial resistance) in the global vaccination strategy.

Typhoid fever, also known as enteric fever, is an acute systemic infectious disease that has an estimated global incidence of 12–22 million cases annually, resulting in 130000–220000 deaths [1–3]. The majority of the disease burden is focused in low- and middle-income settings, mainly Asia and sub-Saharan Africa [1, 4, 5]. Typhoid fever is caused by infection with *Salmonella enterica* serovar Typhi (*S.* Typhi), transmitted through ingestion of food or drink contaminated by human feces containing these bacteria.

Historically, no global public health strategy has been implemented or scaled for control of typhoid fever in resource-poor settings. In settings of endemicity, typhoid fever is managed clinically through both formal and informal healthcare systems instead of through regular preventive vaccination or other vertical health programming. While fluoroquinolones (eg, ciprofloxacin and ofloxacin) have been the first-line antibiotic therapy, the rise in antimicrobial resistance common in South Asia has made alternative antibiotics necessary [6, 7]. As antimicrobial resistance increases and available treatments become inadequate, there is concern that morbidity and mortality will rise concomitantly, underscoring the importance of considering new control strategies for typhoid fever [8, 9].

While typhoid vaccines have been available for decades, important limitations have precluded their widespread adoption in settings of endemicity. The multidose live oral Ty21a and single-dose parenteral Vi polysaccharide have limited efficacy and provide a relatively short duration of protection [10, 11]. Further, while prior studies have found the polysaccharide vaccines to be cost-effective in some scenarios, these vaccines are not approved for use in children aged <2 years (which would allow for integration into routine immunization activities for children through the Expanded Program on Immunization [EPI]) [10–13]. The Ty21a in its current capsular formulation is not approved for use in children aged <5–6 years, depending on jurisdiction [14].

New Vi conjugate vaccines are promising and have many potential advantages over prior vaccines, including higher efficacy and longer duration of protection [15, 16]. Unlike prior typhoid vaccines, conjugate vaccines—which include a number of different versions—have demonstrated safety and immunogenicity in young children with variable dosing, thus allowing for integration within the EPI framework of routine vaccination of children [15–17]. However, Vi conjugate vaccines have yet to be scaled or integrated into any global, national, or subnational strategy, and subsequent decision-making will need to consider these benefits alongside the costs of these new vaccines.

While Vi conjugate vaccines offer a promising intervention to avert disease throughout low- and middle-income typhoid-endemic countries, the conditions and programmatic delivery strategies under which they should be delivered remain unclear. To address this need, we modeled typhoid transmission dynamics, vaccine costs, and outcomes across a range of settings to identify strategies and epidemiologic conditions under which Vi conjugate vaccines would be most cost-effective in settings of endemicity.

## METHODS

### Methods Overview

We developed a mathematical model for transmission of *S.* Typhi informed by previous models [18, 19] and simulated 2 primary interventions with a Vi conjugate vaccine over a 10-year period. This time period was chosen to fully capture the differential benefits of vaccination strategies and duration of immunity while maintaining a shorter time horizon to be conservative given uncertainties in vaccine characteristics, although alternative time horizons were evaluated. We tested 2 primary strategies: (1) routine immunization of infants (aged <1 year) through the EPI continuously over the 10-year period and (2) routine immunization of infants through the EPI with addition of a 1-time catch-up campaign in school-aged children (aged 5–14 years) implemented over 1 month. The primary strategies were selected in informal consultation with experts. In sensitivity analysis, we also tested 2 additional strategies that included EPI immunization with expanded catch-up campaigns in groups aged 1–14 years or 1–30 years. The goal of vaccination was principally focused on reduction of disease burden rather than elimination of transmission. The model assumed 85% coverage for the EPI strategy, based on vaccine coverage estimates in South Asia [20, 21], and 75% coverage for the school catch-up campaign, based on primary and secondary school enrollment estimates for the region [22]. We calibrated the model to the age-stratified population incidence, using a generalized relationship between the age distribution of cases, and to the population incidence, using global empirical data from low and middle-income countries [2]. We tested a range of incidence rates from 10 to 1000 annual cases/100000, where incidence was defined as the true number of underlying clinically symptomatic (rather than culture-confirmed) cases per year, without adjustment for observation from different surveillance or healthcare systems.

### Model of *S*. Typhi Transmission and Vaccine Interventions

**Figure 1.**
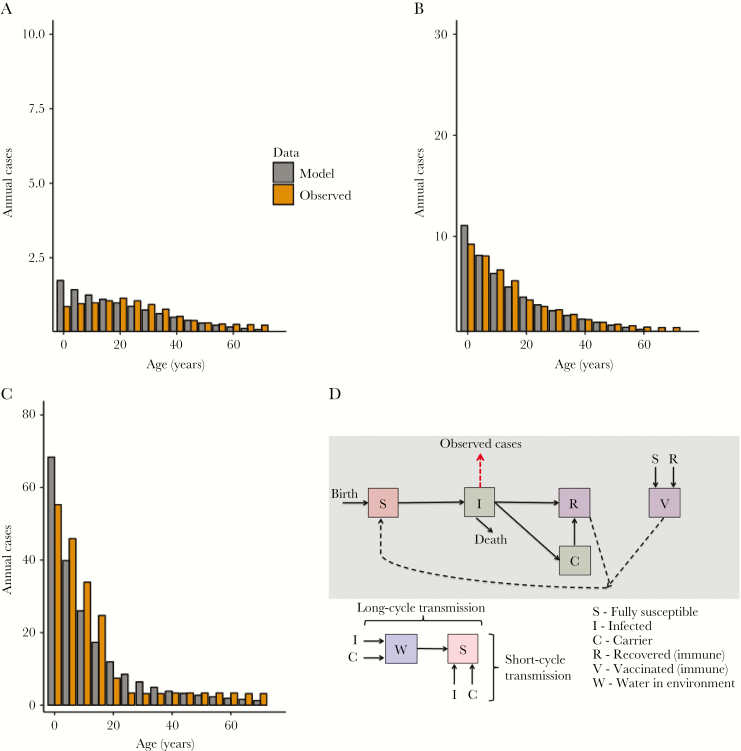
Calibration of transmission model to low-, moderate-, and high-endemicity settings, with model schematic. The model was calibrated to age-stratified annual incidence in 3 scenarios: low endemicity (10 cases/100000; *A*), moderate endemicity (50 cases/100000; *B*), and high endemicity (200 cases/100000; *C*). The model-predicted (gray) and observed (orange) incidences of typhoid cases are shown by age group. Note the different scale for the *y*-axis on each plot. *D*, The model structure for typhoid transmission.

We implemented a dynamic, age-structured, and deterministic compartmental model to simulate transmission of *S.* Typhi over 10 years. We constructed the model to include many important aspects of the natural history of typhoid fever, including subclinical infection, long-term carriage, and waning immunity (Figure 1D). The model structure was similar to that of previously published models, although we did not include partial immunity, based on limited clinical data to support this addition [18, 19]. We tested multiple model structures and followed the rule of parsimony when more-complex models were not identifiable. For those exposed to *S.* Typhi, fully susceptible individuals became infected, contributed to ongoing transmission, and had increased mortality risk associated with infection. We modeled a fraction of infected people to be clinically symptomatic and assumed that the remainder of acutely infected people presented with a subclinical or mild episode of typhoid fever. We defined annual incidence based on the true number of clinically symptomatic people in the population per year, without adjustment for observation from different surveillance or healthcare systems. We used an age-stratified risk of becoming a long-term carrier for all acutely infected people (symptomatic and subclinical), based on empirical data (Appendix) [23]. We assumed an average carriage duration of 10 years, to account for both short-term and long-term carriage and the likelihood for antibiotic exposure over time that would resolve the carrier state; we calibrated a differential relative infectiousness of carriers, given their intermittent shedding, to account for uncertainty in carrier contribution upon transmission and to relax reliance on the estimated average duration of carriage. We tested alternative values through sensitivity analysis, to simulate various possible scenarios. Our model treated people who survived natural infection or recovered from long-term carriage as fully immune, with an exponential distribution applied to immunological waning back to susceptibility, based on the mean duration of immunity that was calibrated. We modeled transmission through 2 routes, following previous models [18]: (1) “short-cycle” transmission from actions in the immediate environment, such as ingestion of contaminated food or drink; and (2) “long-cycle” transmission through drinking contaminated water from the public supply. All 3 infectious states (ie, symptomatic infection, subclinical infection, and carriage) contributed to transmission. We modeled demographic factors, including birth and age-stratified mortality rates [24]. The model stratified the human population by age group (<1 year and subsequent 5-year bins) and dynamically tracked the proportion of the population in each compartment over time to represent the changing incidence over time. We explicitly modeled a single environmental water reservoir to simulate contamination of the public drinking water for long-cycle transmission [18, 25, 26].

Using specified incidence settings and associated age-distribution of cases [2], we calibrated the model to various scenarios, using Bayesian Markov chain Monte Carlo (MCMC) parameter-fitting methods (Figure 1, Table 1, and Appendix) [27]. There is substantial uncertainty in many aspects of transmission and biology of *S.* Typhi, and we used these Bayesian MCMC methods to infer estimates and distributions of possible values for these unknown model inputs. We fitted model parameters for the short-cycle and long-cycle transmission coefficients, fraction of true symptomatic cases, rate of waning immunity, and relative infectiousness of carriers. The remainder of parameters was sourced from literature on epidemiology and natural history (Table 1). We presented the model calibration and key results for 3 example scenarios of low (10 cases/100000), moderate (50 cases/10000), and high (200 cases/100000) endemicity but simulated many different settings within and outside this range of incidences [2].

**Table 1.** Baseline Cohort, Natural History, and Intervention Parameters

Parameter	Base Case Value	Reference(s)
Baseline cohort characteristics		
Population size. no.	100000	…
Annual birth rate, births/1000	21.2	[22]
Mortality rate	Age stratified	[47]
Fixed natural history parameters		
Duration of infectiousness, wk	3	[48]
Long-term carriage, age stratified, %	0.3–10.1	[23]
Duration of carriage, y^a^	10	[23, 49, 50]
Case-fatality rate, symptomatic infection, %	0.5	[28]
Duration of Salmonella infectiousness in environment, wk	3	[18, 51]
Fitted natural history parameters		
Short-cycle transmission coefficient	See appendix	Fitted
Long-cycle transmission coefficient	See appendix	Fitted
Fraction of reporting symptomatic cases	See appendix	Fitted
Relative infectiousness of long-term carriers	See appendix	Fitted
Rate of immunological waning	See appendix	Fitted
Intervention parameters of vaccination strategies		
Coverage, EPI, %	85	[20, 21]
Coverage, school-based program, %	75	[22]
Vaccine efficacy, %	91.5	[15]
Vaccine, mean duration of protection, y	19.2	[15, 16, 18]

^**a**^Duration of carriage includes short-term (<1 year) and long-term carriage, while also accounting for concurrent antibiotic use over time, which would cure a carriage state.

The model treated vaccination with a Vi conjugate vaccine as an all-or-nothing intervention, meaning that a proportion of vaccinated individuals became fully immune to infection, while the remainder did not mount a sufficient immune response and remained susceptible. The vaccine intervention was evenly distributed across all model states (eg, susceptible, recovered, and carrier) since a vaccination program would not be aware of who was already immune because of past natural infection, although only affected susceptible individuals who would benefit from vaccination. We used data from clinical trials on Vi conjugate vaccines to estimate the duration of immunity; we extrapolated the rate of serological waning in the vaccinated cohort from the trial data, with an assumption that this waning was exponentially distributed as per common modeling practice [15, 16, 18]. Vaccine efficacy was estimated based on clinical trial data [15].

### Cost-effectiveness Model and Assumptions

We estimated the cost and averted disability of Vi conjugate vaccination strategies to calculate their cost-effectiveness under various conditions. We modeled disability following published sequelae and disability weights for typhoid fever (Table 2) [28, 29]. We used the conventional disability-adjusted life-year (DALY), which is a measure of disease burden for 1 year of human life, combining the burden of premature mortality (ie, years of life lost) and morbidity (ie, years lived with disability from disease), where 0 is perfect health and 1 is the equivalent to losing a year of healthy life. For all symptomatic cases, we calculated age-specific case-years and subsequently distributed these case-years across 4 possible sequelae: moderate acute infectious disease, severe acute infectious disease, abdominal pain and distension (including a subset with intestinal perforation), and gastrointestinal bleeding (Table 2) [28, 29]. Each sequela was assigned a corresponding disability weight over the course of a standard symptomatic period of 3 weeks. We estimated a case-fatality rate of 0.5% for symptomatic cases across all ages, assuming that each received treatment on the basis of published literature and potential biases of hospital-based studies [28], although we also varied this parameter to account for uncertainty, variability between countries, and trends in increasing antimicrobial resistance that may affect the case-fatality rate. We computed years of life lost with World Health Organization life tables for Southeast Asia [24].

**Table 2.** Cost and Disability Model Parameters

Parameter	Cost, 2016 $	Unit	Comment	Reference(s)
Direct costs				
Conjugate Vi typhoid vaccine	2.50	Per vaccine schedule	One-dose schedule	[35]
EPI delivery (ages <1 y)	1.10	Per dose	One delivery	[30, 32–34]
School-based delivery (ages 5–14 y)	1.30	Per dose	One delivery	[31, 32]
Community-based delivery (ages 1–4 and >14 y)	1.50	Per dose	One delivery	[31, 32]
Indirect costs				
Child (<15 y), outpatient	33.00	Per patient	…	[36]
Adult (≥15 y), outpatient	66.00	Per patient	…	[36]
Child (<15 y), inpatient	222.00	Per patient	9% admitted	[36]
Adult (≥15 y), inpatient	444.00	Per patient	27% admitted	[36]
Health state	Percentage of Case-Years (95% CI)	Disability Weight (95% CI)		
Acute infectious disease, moderate	35.0 (26.0–44.3)	0.051 (.032–.074)		[28, 29]
Acute infectious disease, severe	47.75 (38.0–57.4)	0.133 (.088–.19)		[28, 29]
Abdominal pain and distension (including intestinal perforation)	17.0 (10.0–25.7)	0.324 (.22–.442)		[28, 29]
Gastrointestinal bleeding	0.25 (0–2.0)	0.325 (.209–.462)		[28, 29]

Abbreviations: CI, confidence interval; EPI, Expanded Program on Immunization.

Intervention costs (in 2016 dollars) included direct and indirect sources and were computed from both the perspective of society and a national vaccination program. Direct programmatic costs included the cost of vaccine production, transportation, storage, wastage (10%), vaccination supplies, delivery, training, and worker salaries [30–34]. We assumed a vaccine cost (1-dose schedule) of $2.50, based upon recent pricing of comparable rotavirus vaccinations [35], although because the final cost may change, we tested other possible values in our sensitivity analyses. We estimated vaccination delivery costs for the EPI and for a one-time school-based catch-up campaign on the basis of published vaccine costing studies (eg, rotavirus and pneumococcal vaccine for integration into the EPI and human papillomavirus vaccine for use in school-based programs) [30–34]. We used costing data from global vaccination programs in low- and middle-income countries since programmatic delivery for many vaccines is comparable [30–34]. We estimated a total per-dose delivery cost of $1.10 in the EPI (recipient age, <1 year), $1.30 in a school-based program (5–14 years), and $1.50 for community-based programs (1–4 years and >14 years), where each child or adult received 1 vaccination to complete the schedule [30–34]. For societal cost estimations, we accounted for the cost of illness for outpatient and inpatient settings on the basis of prior costing literature, including both inpatient and outpatient healthcare utilization costs focused in South Asia [36].

The cost-effectiveness of each vaccination intervention was quantified by the incremental cost-effectiveness ratio (ICER; dollars per DALY averted). The ICER compares 2 strategies and is computed as the difference in cost between strategies divided by the difference in averted DALYs. We assumed a base case intervention of no vaccination since this is the current widespread practice. We defined strategies as highly cost-effective if the ICER was less than the per capita income for countries designated by the World Bank as low-income countries (ie, $1035) [22, 37]. However, to relax reliance on a single willingness-to-pay threshold, we generated a cost-effectiveness acceptability curve that provides data across a wide range of potential per capita incomes (ie, willingness-to-pay thresholds). Total costs and disability were discounted at 3% annually as per common practice, although undiscounted results were also computed [38]. We defined an incidence threshold for each vaccination strategy, such that vaccination strategies with an incidence above this threshold were considered highly cost-effective.

### Sensitivity and Uncertainty Analysis

We tested the robustness of our primary study findings with scenario, 1-way sensitivity, and uncertainty analyses that varied key model parameters across a range of plausible values. The 1-way sensitivity analysis tested the effect of one model parameter on the ICER of each strategy, including different vaccine costs (eg, 2-dose schedule), case-fatality rates from typhoid fever, and a full range of willingness-to-pay thresholds. We also provided a conservative scenario analysis with lower vaccine efficacy (80%), shorter duration of immunity (10 years), and higher carrier contribution. We tested the inclusion of expanded societal benefits for vaccine-averted typhoid fever [39, 40]. These included productivity losses due to days of work missed from illness or caring for children and cost savings from reduced antibiotic use (Supplementary Table 4) [39–41]. Other possible benefits could be increased educational attainment in children, reductions in antibiotic resistance, or increased foreign investment and tourism, but these factors were not included, owing to a lack of existing data to quantify such benefits.

We used an uncertainty analysis to generate a 95% uncertainty interval (UI) around the ICER of each strategy, by simultaneously varying multiple key model parameters for typhoid transmission, vaccination, and cost-effectiveness (Appendix). The 95% UI included sampling of different transmission trajectories from the posterior distribution of the MCMC parameter fits. The model code is available online [42].

## RESULTS

### Epidemiology of Typhoid Fever

The fitted model reproduced the age distribution of incidence of typhoid fever for each example scenario of low endemicity (10 cases/100000), moderate endemicity (50 cases/100000), and high endemicity (200 cases/100000); the proportion of cases in younger age groups increased in higher-incidence settings (Figure 1). We measured the disease burden at baseline for each scenario in a population of 100000 people aged >10 years; we estimated 12 DALYs in a low-endemicity setting, 63 DALYs in a moderate-endemicity setting, and 260 DALYs in a high-endemicity setting (Supplementary Table 1). We found that the majority (>95%) of DALYs were attributable to mortality from typhoid fever.

### Population Effectiveness of EPI and EPI With Catch-up Vaccination

**Figure 2.**
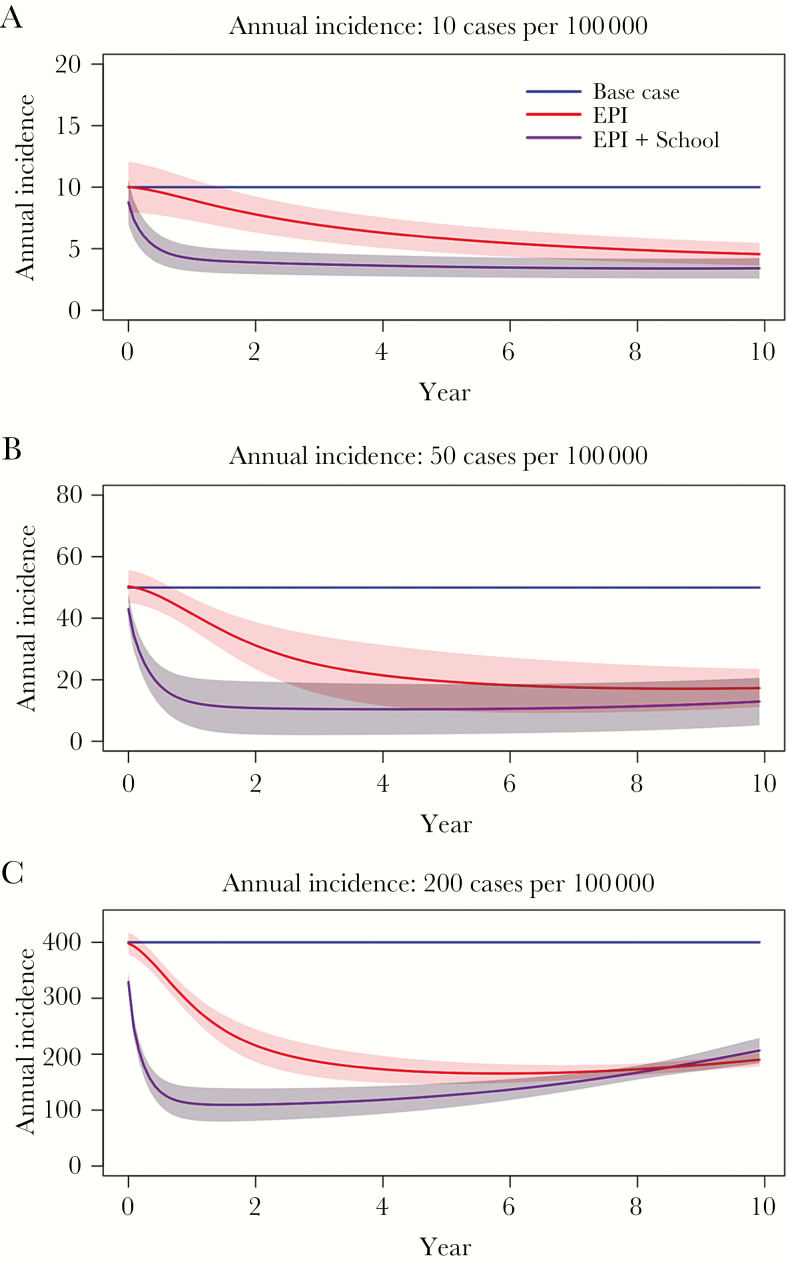
Effectiveness of Vi conjugate vaccination strategies on typhoid fever incidence. We modeled the effectiveness of routine immunization of infants (through the Expanded Program on Immunization [EPI]) and routine immunization through the EPI with 1 catch-up campaign in school-aged children (EPI+Catch-up) on the incidence of typhoid fever over a 10-year period. We modeled settings of low endemicity (10 cases/100000; *A*), moderate endemicity (50 cases/100000; *B*), and high endemicity (200 cases/100000; *C*). Note the different scale for the *y*-axis on each plot.

Under the base case scenario, routine immunization through the EPI resulted in a gradual reduction in population incidence, ultimately reaching a plateau in incidence reduction (Figure 2). The combined strategies (eg, routine immunization through the EPI plus a one-time catch-up campaign) produced larger and faster reductions in incidence but saw infection rebound over time in the moderate- and high-endemicity setting (Figure 2). No vaccination strategy resulted in elimination of typhoid fever.

### Cost-effectiveness of EPI and EPI With Catch-up Vaccination

When assuming a 0.5% case-fatality rate from clinically symptomatic typhoid fever, the Vi conjugate typhoid vaccine was highly cost-effective by routine immunization through EPI in settings with an annual incidence of >50 (95% UI, 40–75) cases/100000 and for the combined strategy (routine immunization through the EPI plus a one-time catch-up campaign in children aged 5–14 years) in settings with an annual incidence of >130 (95% UI, 50–395) cases/100000. In the conservative scenario, the Vi conjugate typhoid vaccine was highly cost-effective by routine immunization through EPI in settings with an annual incidence of >70 cases/100000 and for the combined strategy (routine immunization through the EPI plus a one-time catch-up campaign in children aged 5–14 years) in settings with an annual incidence of >180 cases/100000. When assuming direct programmatic costs alone, we found that the EPI strategy was highly cost-effective in settings with an incidence of >70 (95% UI, 45–125) cases/100000 and for the combined strategy (routine immunization through the EPI plus a one-time catch-up campaign) in settings with an incidence of >200 (95% UI, 95–550) cases/100000.

We tested an additional set of strategies, including expanded catch-up campaigns, in the model. In this scenario analysis, we found that routine immunization through the EPI plus a one-time catch-up campaign was highly cost-effective in children aged 1–14 years with an annual incidence of >110 cases/100000 and in people aged 1–30 years with an annual incidence of >350 cases/100000. Notably, routine immunization through the EPI plus a one-time catch-up campaign in school-aged children (age 5–14 years) was dominated in this scenario, while routine immunization through the EPI alone remained unchanged.

**Figure 3.**
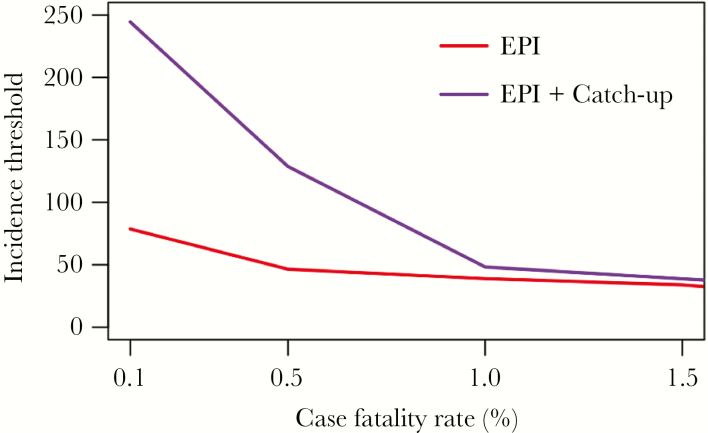
Scenario analysis for the cost-effective incidence threshold under changing case-fatality rate estimates. This scenario analysis tested the effect of increasing the case-fatality rate, which may result from increasing prevalence of antimicrobial resistance to *Salmonella enterica* serovar Typhi.

**Figure 4.**
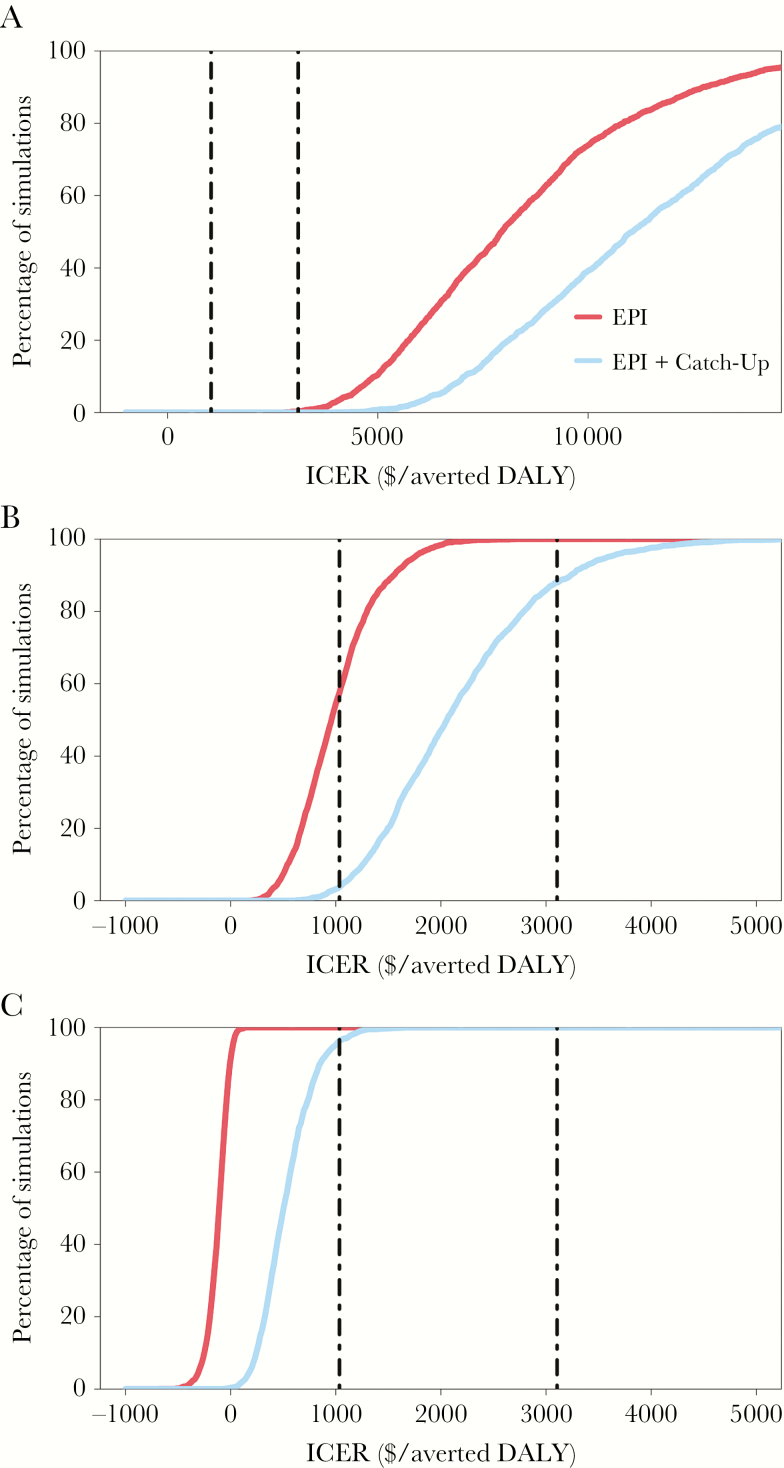
Cost-effectiveness acceptability curves assessing the probability either strategy falls below the willingness-to-pay threshold. We used a probabilistic sensitivity analysis varying all cost, disability, and epidemiology model inputs to compute the probability of each strategy being highly cost-effective at a specified willingness-to-pay threshold ($/averted DALY). Curves are provided for 3 settings: low endemicity (10 cases/100000; *A*), moderate endemicity (50 cases/100000; *B*), and high endemicity (200 cases/100000; *C*). The vertical lines indicate 2 commonly used willingness-to-pay thresholds of 1 times the gross domestic product (GDP) per capita ($1035/averted DALY; left) and 3 times the GDP per capita ($3105/averted DALY; right). A negative incremental cost-effectiveness ratio (ICER) reflects a cost-saving strategy. Note that the low-incidence setting (*A*) is graphed across a wider *x*-axis scale as compared to settings of moderate and high endemicity.

**Figure 5.**
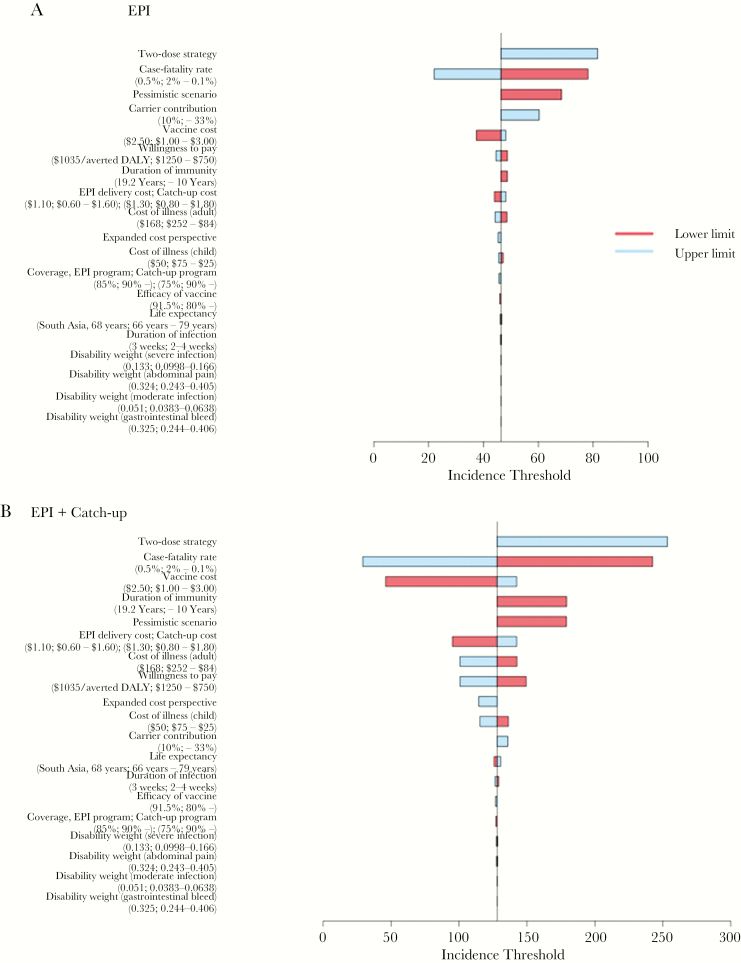
One-way sensitivity analysis of key model parameters to assess changes in the incidence threshold for either strategy. This analysis tested the effect of varying single model parameters for cost, disability, and epidemiology on the cost-effective incidence threshold for vaccination through the Expanded Program on Immunization (EPI) relative to no vaccination (*A*) and routine immunization through the EPI with a school-based catch-up campaign relative to routine immunization through the EPI alone. The horizontal axis depicts the incidence (cases/100000) at which the strategy was highly cost-effective (willingness to pay of $1035/averted disability-adjusted life-year [DALY]). Each model parameter is presented with the base case value, and lower and upper range in parentheses.

The incidence threshold was highly sensitive to the typhoid-related case-fatality rate, which contributed the majority of the disease burden. As the case-fatality increased, both vaccination strategies became more cost-effective over time (Figure 3A). Correspondingly, increasing the case-fatality rate from 0.5% to 1% reduced the incidence threshold for routine vaccination through EPI from 50 to 40 cases/100000, while a reduced case-fatality rate of 0.1% increased the threshold to 80 cases/100000; the effect of case-fatality rate on routine immunization through the EPI plus a one-time catch-up campaign were even more influential. We computed cost-effectiveness acceptability curves to examine the relationship between cost-effective incidence thresholds for the 2 strategies in relation to willingness-to-pay thresholds that may be specific to a setting (defined as the gross domestic product per capita of the country; Figure 4). We found that different willingness-to-pay thresholds (defined as the gross domestic product per capita of the country) affected incidence thresholds in the base case scenario (Figure 5).

### Sensitivity Analysis

In 1-way sensitivity analyses, the cost-effective incidence threshold, above which each vaccination strategy was highly cost-effective, were most sensitive to typhoid case-fatality rate, cost of vaccine and delivery, vaccine efficacy and duration of protection, carrier contribution, and willingness-to-pay threshold (Figure 5). In simulations with a longer time horizon, the cost-effectiveness and incremental value of more-aggressive vaccination strategies as compared to routine immunization through the EPI was reduced, which was in part driven by infection rebound. When including larger societal benefits from averted typhoid cases, vaccination strategies were cost-effective at lower incidences.

## DISCUSSION

In this study, we used a dynamic transmission and cost-effectiveness model to identify incidence thresholds above which to use targeted strategies with new Vi conjugate vaccines against typhoid fever. While global estimates for typhoid fever incidence are uncertain, many studies suggest that typhoid fever in South Asia and Africa is highly endemic (>100 cases/100000), although there is considerable within-country variation [1, 2, 4]. When assuming a 0.5% case-fatality rate for clinically symptomatic typhoid fever, we found that routine immunization of infants through the EPI was highly cost-effective in settings with an annual incidence of >50 cases/100000 and that routine immunization through the EPI with additional inclusion of a catch-up campaign in school-aged children (recipient age, 5–14 years) was highly cost-effective in settings with an incidence of >130 cases/100000. These results were highly sensitive to the estimated case-fatality rate, carrier contribution to transmission, and vaccine characteristics, and incidence thresholds were higher under the conservative scenario on these parameters. In settings where the typhoid case-fatality rate is higher, possibly in relation to increasing incidence of antimicrobial-resistant *S.* Typhi or lower access to healthcare, vaccination would be substantially more cost-effective, meaning that lower-incidence settings may consider vaccination.

For routine immunization through the EPI, we found substantial reductions in the incidence in many settings and that this strategy would be highly cost-effective in settings of moderate endemicity. For routine immunization through the EPI plus a catch-up campaign in school-aged children, we found larger reductions in the number of cases and the disease burden, compared with EPI alone. We found that the incidence threshold for cost-effective vaccination through the EPI plus a school catch-up campaign was higher than the incidence threshold for immunization through the EPI alone, although there was greater uncertainty around this estimate. This means that higher-incidence settings should consider adding a one-time catch-up campaign to their typhoid control strategy. Notably, when considering expanded catch-up campaigns, we found that routine immunization through the EPI with an expanded catch-up program (recipient age, 1–14 years) dominated routine immunization through the EPI with a school-based catch-up program (5–14 years) and would be preferred in higher-incidence settings. Additionally, the average age at infection increased following a vaccination campaign. Given an increased risk for long-term carriage at older ages, mass vaccination could possibly contribute to an increased risk of long-term carriage that continues to drive transmission.

The effectiveness results from our study broadly aligned with data from a prior modeling study that simulated the effect of routine immunization in Vellore, India [18]. In particular, this study found that vaccination resulted in substantial indirect benefits to unvaccinated children in the short-term but similarly found a rebound in the number of infections over a longer horizon, although, overall, it provided larger reduction in total cases [18]. In settings where significant rebound of incidence is predicted, additional catch-up campaigns could also be considered. Recently, a cost-effectiveness analysis of conjugate typhoid vaccines administered in 5 low- and middle-income settings similarly found that routine vaccination would be cost-effective but emphasized that the optimal strategy often included a one-time catch-up campaign [43].

A key finding was that the case-fatality rate of typhoid fever was a critical and uncertain parameter that drives the incidence thresholds of various vaccination strategies. Most composite estimates have been in the range of 0.5% to 1%, but this could be biased upward, because most studies are hospital based, or downward, because of unascertained deaths at home, and it also is likely to vary by country and setting. Furthermore, as antimicrobial resistance increases and available treatments become ineffective, there is concern about an associated increase in the case-fatality rate over time. The typhoid case-fatality rate is also driven by setting-specific factors, including access to and quality of healthcare and, possibly, interaction with other infectious diseases (eg, human immunodeficiency virus infection) [44]. To address this uncertainty, we present estimates for incidence thresholds by using a range of case-fatality rates through scenario and sensitivity analysis, which allows for decision-making using different assumptions on case-fatality rate.

We optimized vaccine strategies to the economic willingness-to-pay threshold of a low-income country, although this may be too low or high for some settings. In South Asia, many countries have transitioned into middle-income status and may refine their vaccination decision within this economic context. To support a programmatic decision, we performed a scenario analysis and provided the cost-effectiveness acceptability curves, which provide model results across a wide range of willingness-to-pay thresholds (ie, country-specific economic thresholds) for more-context-specific decision-making. In higher-income countries with correspondingly higher economic thresholds, more-aggressive strategies were more often highly cost-effective than in low-income countries.

In the sensitivity and uncertainty analyses, we varied multiple key model parameters related to the biology of typhoid transmission, vaccination, and cost-effectiveness. We identified some sensitive model inputs, many of which are likely to be setting specific and can be refined within a particular context with data on case-fatality rate, economic status (ie, willingness to pay), vaccine cost, and other epidemiologic factors. Importantly, we generated 95% UIs based on a range and distribution of possible model inputs, and this interval should be conceptualized as a broad range of possible outcomes for a given setting, given assumptions on tested ranges and distribution of parameters. For a 2-dose vaccination schedule, which would double the vaccine cost, we found the incidence threshold to intuitively become higher because of an increased cost of the program. However, broadly these analyses found that vaccination can still be highly cost-effective in settings of moderate endemicity when taking into account uncertainty and variation in the value of certain key parameters.

The goal of this study was to provide incidence thresholds to guide vaccine implementation, but the typhoid incidence itself is often unknown and varies within a country and across time; therefore, surveillance and, perhaps, new strategies are first required to ascertain the typhoid incidence for a given setting before these study results can be used. Furthermore, while we found the typhoid Vi conjugate vaccine to be cost-effective in many settings of endemicity, the total budgetary requirements may still be too large. The aim is to support country prioritization among many competing health needs. Given the within-country heterogeneity in typhoid fever burden [45], countries may consider targeted vaccination campaigns that prioritize high-risk areas (ie, subnational decision-making) in the scale up of vaccination programs, which could help address budgetary constraints.

The findings of this study should be understood within the limitations of the model assumptions, uncertainty in the biology of typhoid transmission, and country-specific heterogeneities. To construct broadly generalizable incidence thresholds, we used an approximate epidemiologic relationship between incidence and the age distribution of cases, and we recognize that this may oversimplify complex setting-specific heterogeneities and uncertainties. Importantly, there is substantial uncertainty in the biology of *S.* Typhi transmission, particularly the relative contribution of short versus long-cycle transmission and long-term carriers, and in vaccine efficacy and duration of protection. To address this, we applied Bayesian model calibration to age-structured data to infer unknown parameters (eg, the relative contributions of the 2 sources of transmission) and generated a set of model inputs to create a 95% UI for all transmission projections; additionally, we performed sensitivity analyses to ensure our primary results were broadly robust. We assumed homogenous mixing within each subpopulation, no migration, exponential waning of immunity, and 1 environmental water reservoir following common modeling practice for waterborne infections, although these factors are more complex in reality [18, 25, 26]. While the final cost of the typhoid Vi conjugate vaccine is not yet known, we assumed a cost that followed the cost of similar recently introduced vaccines (eg, rotavirus vaccine) and tested the effect of differing price through sensitivity analysis. Given our focus on pediatric-based vaccination strategies, these approaches would not address older populations that are exposed to *S.* Typhi, except through the indirect benefits of vaccination. Typhoid vaccines have the potential to provide value outside of health outcomes alone (eg, school and work productivity, future earnings, and tourism) [39, 40]. While these effects are difficult to estimate, this was explored in sensitivity analysis and improved the cost-effectiveness of vaccination, suggesting that future work should examine these potential benefits [39–41]. We did not model seasonality in transmission, although this would not be expected to affect the primary result. We did not include interventions for water, sanitation, and hygiene interventions, although these are likely to be important for sustainable control of typhoid fever and will likely reduce the future need for vaccination. Finally, conjugate vaccines may not be protective against *S.* Paratyphi, which contributes to global enteric fever cases and will require additional intervention to address [46].

The new Vi conjugate typhoid vaccines pose great potential to address typhoid fever in the low- and middle-income countries and have renewed interest in developing a global public health strategy using vaccination. This study supports the introduction of the vaccine as a public health intervention in some settings of endemicity to address the global disease burden of typhoid fever.

## Supplementary Data

Supplementary materials are available at *The Journal of Infectious Diseases* online. Consisting of data provided by the authors to benefit the reader, the posted materials are not copyedited and are the sole responsibility of the authors, so questions or comments should be addressed to the corresponding author.

## Supplementary Material

Supplementary Material

## Notes

***Acknowledgments.*** N.C. L. had full access to all of the data in the study and takes responsibility for the integrity of the data and the accuracy of the data analysis. N. C. L. and J. R. A. designed the study. N. C. L. and R. G. analyzed the data. All authors interpreted findings of data analysis. N. C. L. wrote the first draft of the manuscript. All authors contributed intellectual content and approved final draft of the manuscript.

***Disclaimer.*** The funding organizations had no role in the design and conduct of the study; collection, management, analysis, and interpretation of the data; the preparation, review, or approval of the manuscript; or the decision to submit the manuscript for publication.

***Financial support.*** This work was supported by the Medical Scientist Training Program, Stanford University School of Medicine (support to N. C. L.). This article is part of the supplement “Surveillance of Enteric Fever in Asia Project (SEAP),” sponsored by the Sabin Vaccine Institute. This publication was made possible by the Bill and Melinda Gates Foundation (grant OPP11130007).

***Supplement sponsorship.*** This article is part of the supplement “Surveillance for Enteric Fever in Asia Project,” sponsored by the Sabin Vaccine Institute.

***Potential conflicts of interest.*** All authors: No reported conflicts. All authors have submitted the ICMJE Form for Disclosure of Potential Conflicts of Interest. Conflicts that the editors consider relevant to the content of the manuscript have been disclosed.

## Notes

Presented in part: 10th International Conference on Typhoid and Other Invasive Salmonelloses, Kampala, Uganda, 4 April 2017, Oral abstract; American Society of Tropical Medicine and Hygiene, Baltimore, Maryland, 9 November 2017, Oral abstract 1981.
