# Supplementary material for: Comparison of Strategies and Incidence Thresholds for Vi Conjugate Vaccines Against Typhoid Fever: A Cost-effectiveness Modeling Study
Source: J Infect Dis. 2018 Feb 12;218(Suppl 4):S232–42. doi: 10.1093/infdis/jix598 (PMC6226717; doi:10.1093/infdis/jix598)
Supplement: Supplementary Material [file jix598_suppl_supplementary_material.docx]

**Supplemental Materials**

Lo NC, Gupta R, Stanaway JD, Garrett DO, Bogoch II, Luby SP, and Andrews JR. Comparison of Strategies and Incidence Thresholds for Vi Conjugate Vaccines Against Typhoid Fever: A Cost-effectiveness Modeling Study. Journal of Infectious Diseases (2017).

**Contents**

Section 1: Technical appendix.……………..…………….……….……..………………….Page 3

Section 2: Supplemental figures and tables…………………..…………….………….....…Page 7

- **Section 1: Technical appendix**

In this supplement, we provide additional methodological details on the mathematical transmission model for *Salmonella* Typhi, and the cost-effectiveness analysis for Vi conjugate vaccination.

- *Transmission model for S. Typhi*
- We implemented a dynamic, age-structured, and deterministic compartmental model that simulated transmission of *S.* Typhi in a population over 10 years as described in the Methods. The transmission model was described by a series of ordinary differential equations.

Equation 1 modeled a changing population of susceptible persons (S) over time t:

- $\left[ 1 \right] \frac{dS}{dt}=u_{Bi}-\beta SI-\beta SCr-\lambda SW+\omega R+\omega_{vacc}V-\mu S$
- The population of susceptible persons increased with new births (rate $u_{Bi}$) and people who lost their immunity (rate$\omega$) that were previously in the recovered/immune (R) or vaccinated (V) compartments. The susceptible population decreased when persons were infected via: (1) short-cycle transmission at a rate of $\beta$ in relation to the infected (I) population; (2) short-cycle transmission at a rate of $\beta$ in relation to the carrier (C) population with a relative carrier infectiousness of $r$; or (3) long-cycle transmission at a rate of $\lambda$ in relation to the concentration of infectious bacteria in the environmental water supply (W). We included background age-specific mortality for all compartments of rate $\mu$ (Table A1). Each age group had a separate state compartment, and we modeled interaction between all age compartments.

Equation 2 modeled a changing population of infected persons (I) over time t:

- $\left[ 2 \right] \frac{dI}{dt}=\beta SI+\beta SCr+\lambda SW-\gamma I-\theta I-(\mu+\mu_{i})I$

The population of infected persons increased with new infections from short-cycle (generated by acutely infected and long-term carrier populations) and long-cycle transmission routes. The infected population recovered from their infection at a rate of $\gamma$, where $1/\gamma$ was average duration of infectiousness. A fraction of infected persons became carriers at an age-specific rate of $\theta$ (Ames and Robins, Am J Public Health Nations Health 1943). We included an additional typhoid infection-specific case fatality rate ($\mu_{i}$).

- Equation 3 modeled a changing population of long-term carriers (C) over time t:
- $\left[ 3 \right] \frac{dC}{dt}=\theta I-\nu C-\mu C$
- The population of long-term carriers increased as acutely infected persons became carriers at an age-specific rate of $\theta$ (Table A1). The carrier population recovered from their carriage state at a rate of $\nu$, where $1/v$ was average duration of long-term carriage.
- Equation 4 modeled a changing population of recovered (immune) persons (R) over time t:
- $\left[ 4 \right] \frac{dR}{dt}=\gamma I-\omega R+vC-\mu R$
- The population of recovered (immune) persons increased as acutely infected persons recovered from their infection at a rate of $\gamma$, where $1/\gamma$ was average duration of infectiousness. The recovered population waned from full immunity back to susceptibility at a rate of $w$, where $1/w$was average duration of immunity from natural infection.
- Equation 5 modeled a changing population of vaccinated (immune) persons (V) over time t:
- $\left[ 5 \right] \frac{dV}{dt}=Sa_{vacc}-\omega_{vacc}V-\mu C$
- The population of vaccinated (immune) persons increased as susceptible persons were vaccinated at rate $a_{vacc}$. We modeled the vaccination of susceptible persons at a rate that was equivalent to an annual coverage. The vaccinated population waned from full immunity back to susceptibility at a rate of $\omega_{vacc}$, where $1/\omega_{vacc}$was average duration of immunity from vaccination.

Equation 6 modeled a changing concentration of infectious *Salmonella* in the environment and public water supply (W) over time t:

- $\left[ 6 \right] \frac{dW}{dt}=\xi I+\xi rC-\psi W$
- The concentration of bacteria in the public water supply increased as infected persons and carriers (with relative infectiousness, $r$) excreted infectious bacteria into the environment and public water supply at rate $\xi$. The environmental excretion rate was fixed given its relation to long-cycle transmission rate, which was calibrated. The bacteria degraded in the environment at rate $\psi.$

The model was comprised of age-specific compartments for each state of susceptible, infected, carrier, recovered, and vaccinated. We simulated provinces of 100,000 people for each scenario analysis, although the chosen population size had minimal impact on the model results since the cost-effectiveness of each strategy scales accordingly. We calibrated the model to reach a quasi-equilibrium state of endemic typhoid fever after a burn-in period. In the calibration process, we used Bayesian Markov Chain Monte Carlo (MCMC) methods to estimate the fitted model parameters. In this process, we assumed cases were Poisson distributed and used a time step of one month. The model assumed frequency-dependent transmission given large population and heterogeneous mixing. The model was programmed in R 3.2.3 (R Foundation for Statistical Computing; Vienna, Austria), and the code is available online (see main text reference to Github repository).

- **Table A1: Age-specific model parameters**

| Age groups (years) | Annual mortality rate (per 1,000) | Long-term carriage (%) |
| --- | --- | --- |
| <1 | 34.6 | 0.3 |
| 2-4 | 2.3 | 0.3 |
| 5-9 | 0.8 | 0.3 |
| 10-14 | 0.6 | 0.3 |
| 15-19 | 1 | 0.3 |
| 20-24 | 1.5 | 2.1 |
| 25-29 | 1.7 | 2.1 |
| 30-34 | 2 | 4.4 |
| 35-39 | 2.7 | 4.4 |
| 40-44 | 3.6 | 8.8 |
| 45-49 | 5.3 | 8.8 |
| 50-54 | 7.9 | 10.1 |
| 55-59 | 12.2 | 10.1 |
| 60-64 | 20 | 7.8 |
| 65-69 | 31.6 | 7.8 |
| 70-74 | 50.3 | 7.8 |

| **A**  **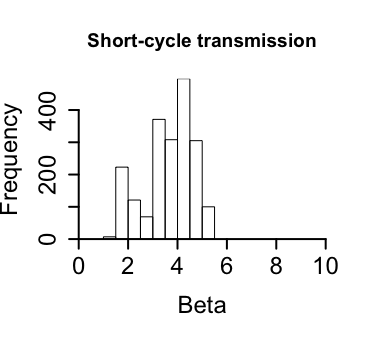** | **B**  **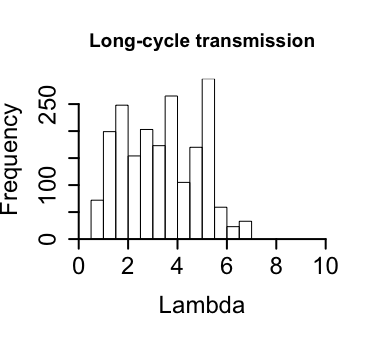** |
| --- | --- |
| **C**  **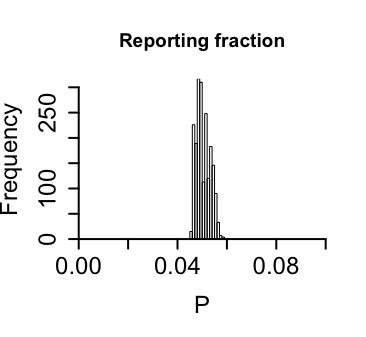** | **D**  **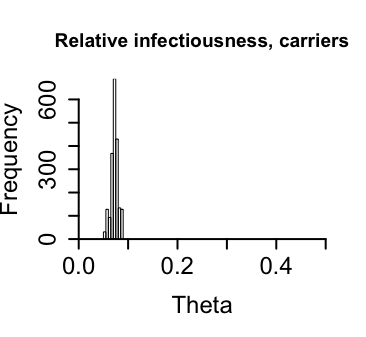** |
| **E**  **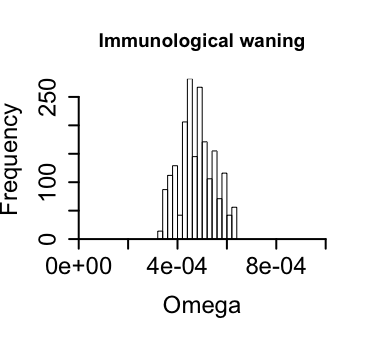** |  |

**Figure A1: Fitted natural history parameters.** Model parameters were calibrated for each incidence scenario. These data are for one model calibration for an incidence scenario of 100 cases per 100,000. We fitted five model parameters: (A) $\beta$, short-cycle transmission coefficient; (B) $\lambda$, long-cycle transmission coefficient; (C) Reporting fraction, fraction of reporting symptomatic cases; (D) $r$, Relative infectiousness of long-term carriers; and (E) $\omega$, rate of immunological waning.

**Section 2: Supplemental figures and tables**

Table S1: Costs, disability, and cost-effectiveness of interventions with a Vi conjugate vaccine against typhoid fever

Table S2: Characteristics of parameter distributions used in probabilistic sensitivity analysis

Table S3: Partial rank correlation coefficients for all model parameters under all tested strategies.

Table S4: Description of expanded societal cost savings for averted typhoid infections

Figure S1: Distribution of incremental cost-effectiveness ratios (ICERs) for settings with varying typhoid fever incidence

Figure S2: Distribution of incidence thresholds for vaccine strategies against typhoid fever using a probabilistic sensitivity analysis

Figure S3: Relationship between cost-effective incidence threshold and willingness-to-pay threshold for typhoid vaccination strategies.

**Table S1: Costs, disability, and cost-effectiveness of interventions with a Vi conjugate vaccine against typhoid fever**

|  | |  | Total costs (2016 US$) | |  | |  | Total disability (DALYs) | |  | | ICER^a^ (US$/DALY) | |
| --- | --- | --- | --- | --- | --- | --- | --- | --- | --- | --- | --- | --- | --- |
| Strategy | | Discounted^a^ | | Undiscounted | | Discounted^a^ | | | Undiscounted | |  | |  |
| *Low endemicity (10 cases per 100,000)* | | | | | |  | | |  | |  | | |
|  | No vaccination | 10,702 | | 12,174 | | 11.9 | | | 26.4 | | -- | | |
|  | EPI | 49,043 | | 55,676 | | 7.6 | | | 16.2 | | 8,879 | | |
|  | EPI with school catch-up | 87,689 | | 94,026 | | 4.4 | | | 9.3 | | 12,102 | | |
|  | EPI with catch-up (1-14 yr.) | 103,482 | | 109,718 | | 3.4 | | | 7.1 | | 16,048 | | |
|  | EPI with catch-up (1-29 yr.) | 162,816 | | 168,851 | | 2.0 | | | 4.1 | | 41,048 | | |
| *Moderate endemicity (50 cases per 100,000)* | | | | | |  | | |  | |  | | |
|  | No vaccination | 48,605 | | 55,290 | | 61.8 | | | 141.7 | | -- | | |
|  | EPI | 637,738 | | 76,361 | | 30.5 | | | 66.7 | | 611 | | |
|  | EPI with school catch-up | 96,712 | | 104,189 | | 14.7 | | | 32.3 | | 1,833 | | |
|  | EPI with catch-up (1-14 yr.) | 109,901 | | 116,949 | | 10.3 | | | 22.3 | | 2,982 | | |
|  | EPI with catch-up (1-29 yr.) | 1696,412 | | 172,886 | | 5.9 | | | 12.8 | | 12,681 | | |
| *High endemicity (200 cases per 100,000)* | | | | | |  | | |  | |  | | |
|  | No vaccination | 156,737 | | 178,396 | | 257.4 | | | 617.9 | | -- | | |
|  | EPI | 131,593 | | 148,710 | | 129.4 | | | 298.0 | | Cost-saving | | |
|  | EPI with school catch-up | 148,363 | | 164,123 | | 88.2 | | | 207.2 | | --^b^ | | |
|  | EPI with catch-up (1-14 yr.) | 149,712 | | 163,648 | | 62.8 | | | 147.4 | | 272 | | |
|  | EPI with catch-up (1-29 yr.) | 195,760 | | 207,489 | | 46.1 | | | 109.6 | | 2,754 | | |

Model estimates computed for a simulated population of 100,000.

DALYs, disability-adjusted life years; ICER, incremental cost-effectiveness ratio

^a^Costs and disability are discounted at 3% annually.

^b^Strategy was dominated (higher cost and less effective, or dominated by extension).

- **Table S2: Characteristics of parameter distributions used in probabilistic sensitivity analysis.**

| **Model parameter** | **Median value** | **Lower bound** | **Upper bound** | **Distribution type** |
| --- | --- | --- | --- | --- |
| Case fatality rate | 0.50% | 0.25% | 1.00% | Triangle dist. |
| EPI delivery cost | $1.10 | $0.60 | $1.60 | Triangle dist. |
| Catch-up cost | $1.30 | $0.80 | $1.80 | Triangle dist. |
| Vaccine cost | $2.50 | $1.00 | $3.50 | Triangle dist. |
| Duration of infection (gastro bleed) | 3 weeks | 1 weeks | 4 weeks | Triangle dist. |
| Duration of infection (abdominal pain) | 3 weeks | 1 weeks | 4 weeks | Triangle dist. |
| Duration of infection (severe) | 3 weeks | 1 weeks | 4 weeks | Triangle dist. |
| Duration of infection (mild) | 3 weeks | 1 weeks | 4 weeks | Triangle dist. |
| Disability weight (gastro bleed) | 0.325 | 0.244 | 0.406 | Triangle dist. |
| Disability weight (abdominal pain) | 0.324 | 0.243 | 0.405 | Triangle dist. |
| Disability weight (severe) | 0.133 | 0.099 | 0.166 | Triangle dist. |
| Disability weight (mild) | 0.051 | 0.038 | 0.064 | Triangle dist. |
| Willingness to pay | $1035.00 | $750.00 | $1250.00 | Triangle dist. |
| Incidence (standard deviation) | 0 | - | - | Normal dist. |
| Life expectancy | 68.43 years | 60.37 years | 76.77 years | Triangle dist. |
| Cost of illness (child) | $50.01 | $25.01 | $75.02 | Triangle dist. |
| Cost of illness (adult) | $168.06 | $84.03 | $252.09 | Triangle dist. |
| Duration of immunity | 19.2 years | 10 years | 19.2 years | Single sided triangle dist. |
| Vaccine efficacy | 91.5% | 80% | 95% | Triangle dist. |
| Carrier contribution | 10% | 10% | 33% | 50%- 10% carrier  25%- 20% carrier  25%- 33% carrier |

Model parameter values picked through Latin-Hypercube sampling.

**Table S3: Partial rank correlation coefficients for all model parameters under all tested strategies.**

| Model parameter | PRCC (A), EPI | PRCC (B), EPI + school catch-up |
| --- | --- | --- |
| Case fatality rate | -0.93 | -0.84 |
| EPI delivery cost  Catch-up cost  Vaccine cost | 0.64  ~0  0.91 | ~0  0.43  0.76 |
| Duration of infection (gastro bleed) | 0.02 | 0.02 |
| Duration of infection (abdominal pain) | -0.03 | -0.02 |
| Duration of infection (severe) | -0.06 | -0.02 |
| Duration of infection (mild) | -0.01 | ~0 |
| Disability weight (gastro bleed) | 0.01 | 0.03 |
| Disability weight (abdominal pain) | -0.02 | ~0 |
| Disability weight (severe) | 0.01 | 0.02 |
| Disability weight (mild) | ~0 | ~0 |
| Willingness to pay | -0.66 | -0.48 |
| Incidence | -0.15 | 0.77 |
| Life expectancy | -0.22 | -0.12 |
| Cost of illness (child)  Cost of illness (adult) | -0.35  -0.55 | -0.25  -0.26 |

Partial rank correlation coefficients (PRCC) quantify the correlation between two variables, while holding all over variables constant. The coefficient is bounded (-1, 1), where a negative value indicates an inverse relation and a positive value indicates a positive relation.

**Table S4: Description of expanded societal cost savings for averted typhoid infections**

| **Parameter** | **Median value** | **Low value** | **High value** |
| --- | --- | --- | --- |
| *Productivity gains^1-3^* |  |  |  |
| Lost days of work | 3.76 days | 2 days | 6 days |
| GDP per year | $1035 | $750 | $1250 |
| Paid days of work per year | 261 days | - | - |
| Lost days of work for caretaker (adult infection) | 1.2 days | 0.2 days | 2.2 days |
| Lost days of parental work  (child infection) | 4.88 days | 3 days | 7 days |
| Proportion of families with caretaker | 90% | 70% | 99% |
| Total (child) | $17.13 | $7.63 | $29.67 |
| Total (adult) | $16.99 | $5.44 | $34.66 |
| *Antibiotic savings^4,5^* |  |  |  |
| Number of extra antibiotic prescriptions^a^ | 3 per infection | 1 per infection | 5 per infection |
| Cost of fluoroquinolones | $0.14 | $0.06 | $0.23 |
| Number of doses | 12 | 10 | 14 |
| Total | $5.04 | $0.60 | $16.10 |
| *Total* |  |  |  |
| Child | $22.17 | $8.23 | $45.77 |
| Adult | $22.03 | $6.04 | $50.76 |

^a^Many patients with febrile illness in typhoid endemic regions are suspected to have typhoid fever, and are treated with antibiotics (e.g. 3 non-typhoid patients for every 1 typhoid patient). If a vaccine program sufficiently reduced typhoid, many patients without typhoid fever would not be unnecessarily treated with antibiotic therapy with associated cost savings.

**A**


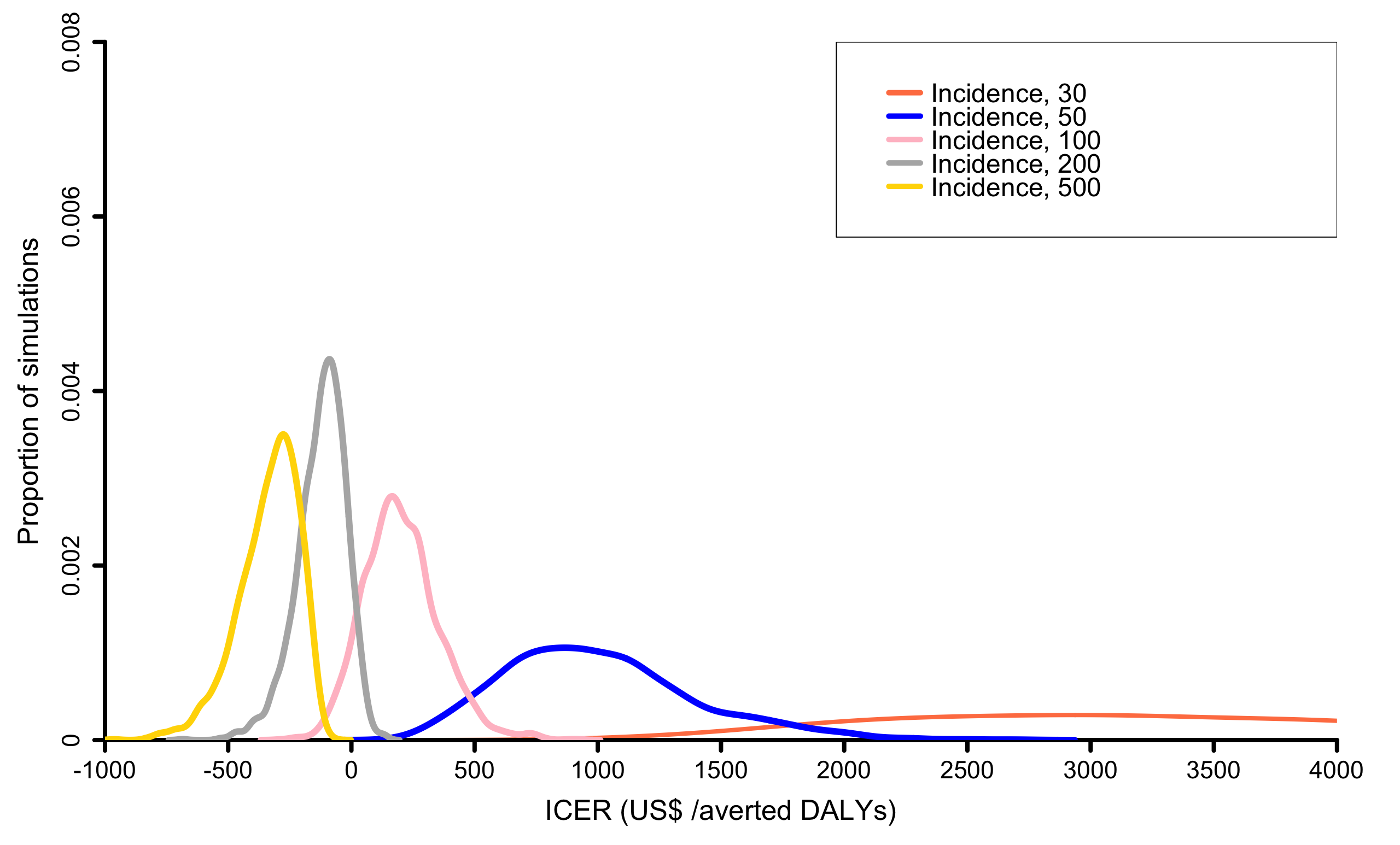


**B**

**
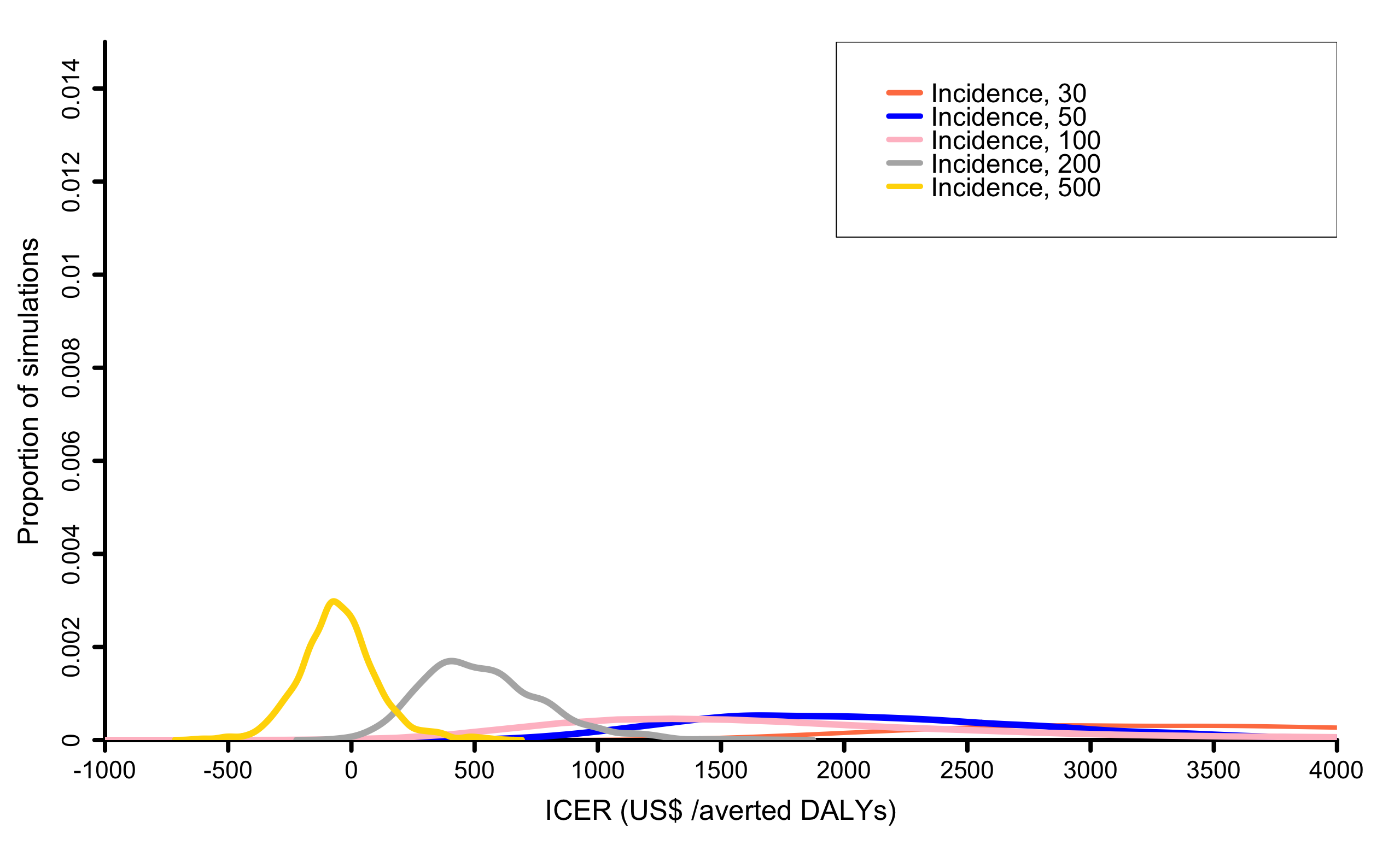
**

**Figure S1: Distribution of incremental cost-effectiveness ratios (ICERs) for settings with varying typhoid fever incidence.** We performed a probabilistic sensitivity analysis to vary multiple model inputs simultaneously (see Table S2) to generate the ICER of (A) routine immunization of infants (EPI) relative to no vaccination; and (B) routine immunization through EPI with one catch-up campaign in school-aged children (EPI+catch-up) relative to EPI alone. Negative ICERs indicate a cost-savings intervention.

**A**

**B
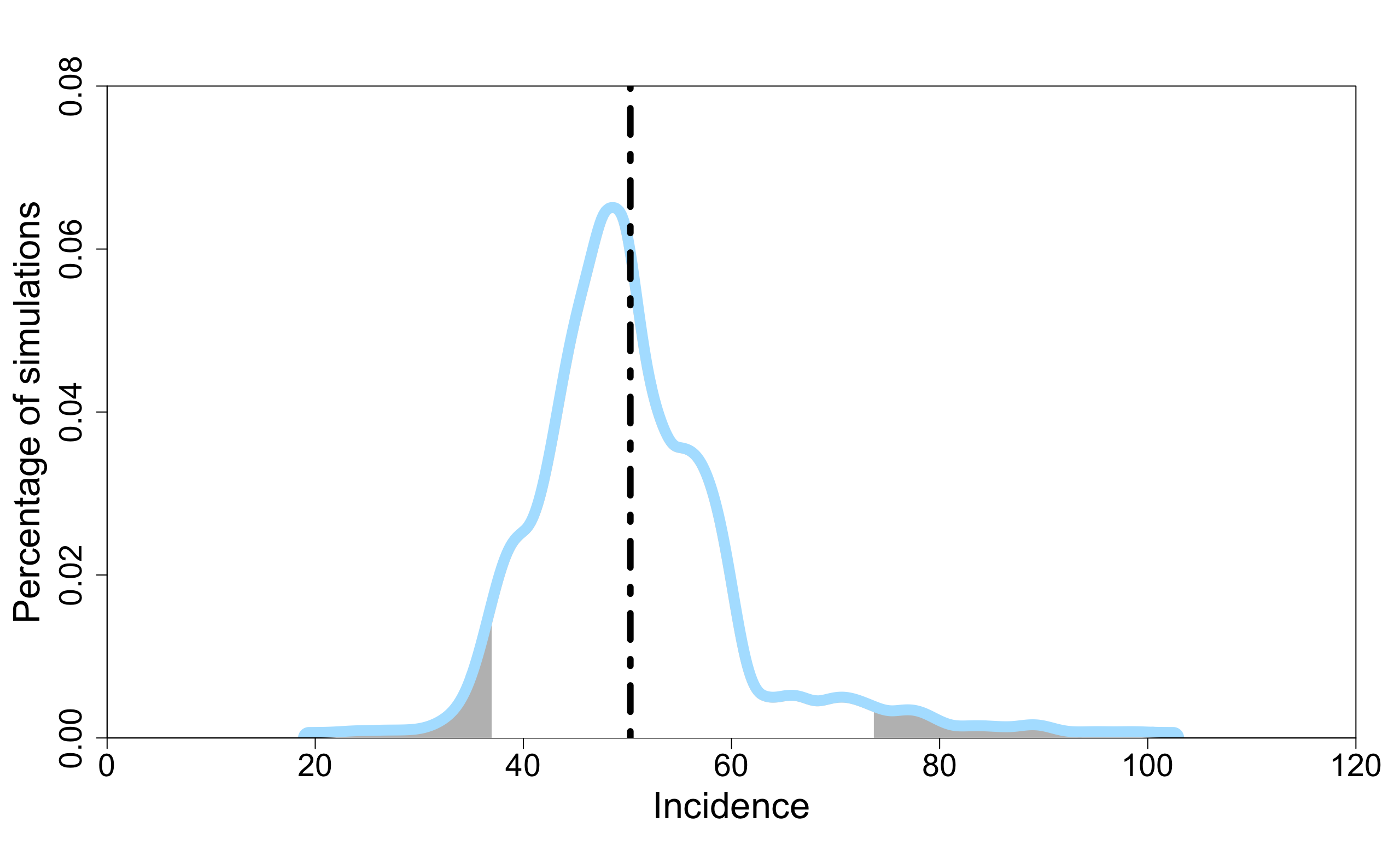

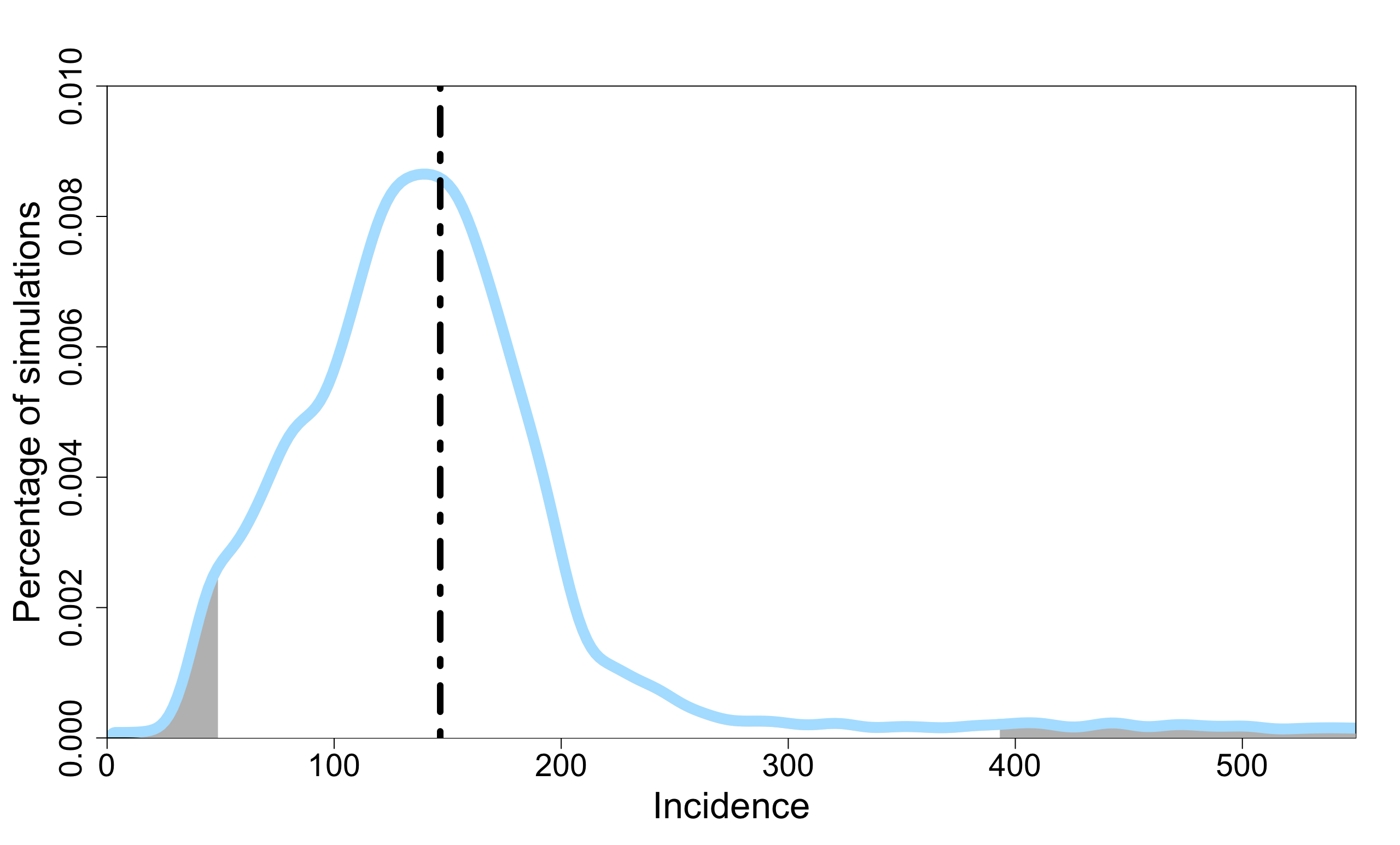
**

**Figure S2: Distribution of incidence thresholds for vaccine strategies against typhoid fever using a probabilistic sensitivity analysis.** We performed a probabilistic sensitivity analysis to vary multiple model inputs simultaneously (see Table S2) to generate a range of possible incidence thresholds for (A) routine immunization of infants (EPI); and (B) routine immunization through EPI with one catch-up campaign in school-aged children (EPI+catch-up). Regions shaded in grey indicate the lower and upper limit (5% total) of simulations tested. The unshaded regions capture the 95% uncertainty interval. Note difference in scale of x-axis between (A) and (B) due to varying widths of incidence threshold distribution.


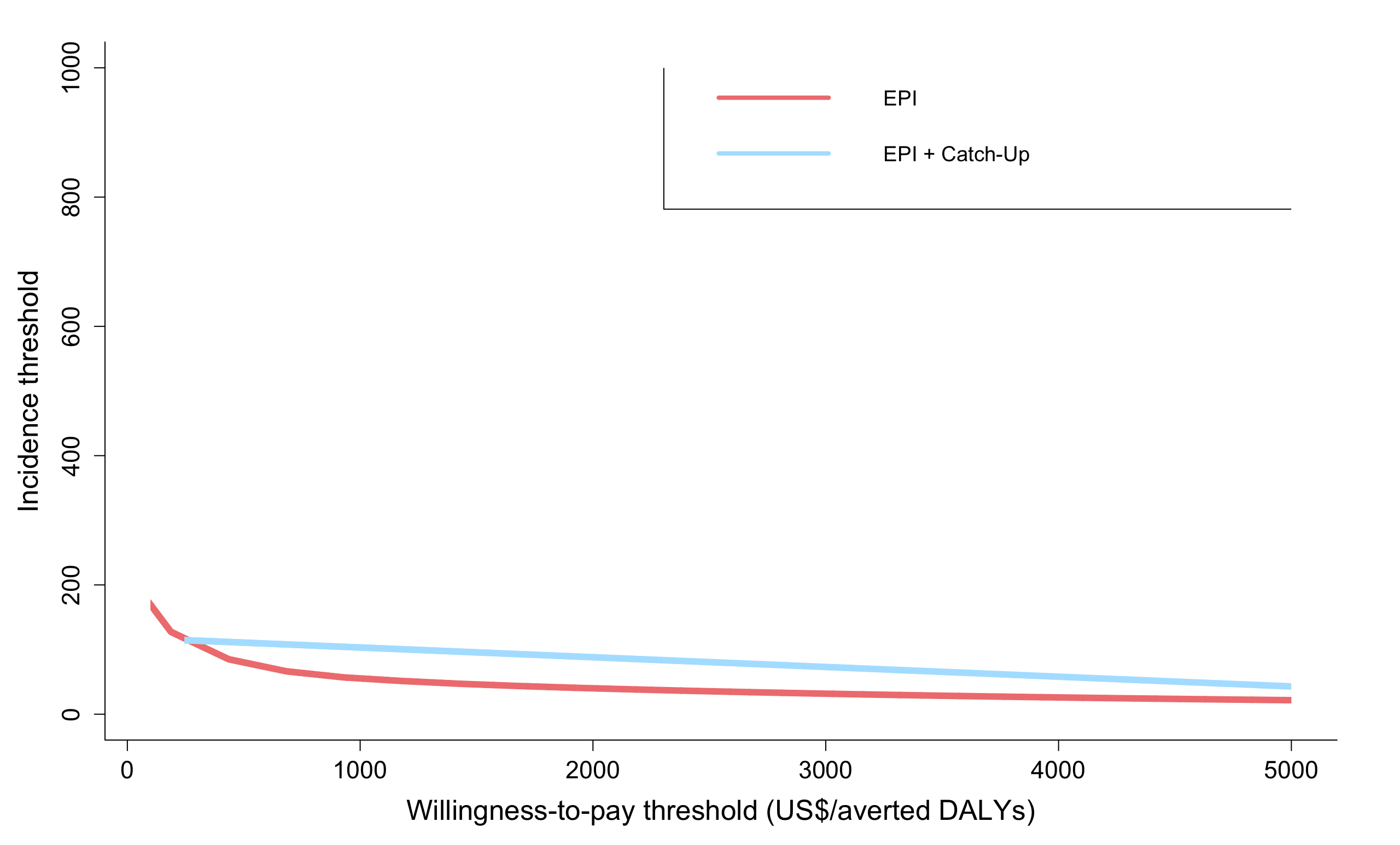


**Figure S3: Relationship between cost-effective incidence threshold and willingness-to-pay threshold for typhoid vaccination strategies.** We computed the base case analysis for incidence threshold for varying willingness-to-pay thresholds (US$/averted DALY).

**References**

1. Kaljee LM, Pach A, Garrett D, Bajracharya D, Karki K, Khan I. Social and Economic Burden Associated With Typhoid Fever in Kathmandu and Surrounding Areas: A Qualitative Study. *J Infect Dis* 2017.

2. Poulos C, Riewpaiboon A, Stewart JF, et al. Cost of illness due to typhoid fever in five Asian countries. *Trop Med Int Health* 2011; **16**: 314-23.

3. Sur D, Chatterjee S, Riewpaiboon A, Manna B, Kanungo S, Bhattacharya SK. Treatment cost for typhoid fever at two hospitals in Kolkata, India. *J Health Popul Nutr* 2009; **27**: 725-32.

4. Chawan VS, Gawand KV, Badwane SV. Fluoroquinolones in India—Are we prescribing it right: A cost variation study. *Natl J Physiol Pharm Pharmacol* 2015; **5**.

5. Andrews JR, Vaidya K, Bern C, et al. High Rates of Enteric Fever Diagnosis and Lower Burden of Culture-Confirmed Disease in Peri-urban and Rural Nepal. *J Infect Dis* 2017.
